# Long-Term Stability of One-Inch Condenser Microphones Calibrated at the National Institute of Standards and Technology

**DOI:** 10.6028/jres.120.012

**Published:** 2015-08-17

**Authors:** Randall P. Wagner, William F. Guthrie

**Affiliations:** National Institute of Standards and Technology, Gaithersburg, MD 20899 USA

**Keywords:** calibration, condenser microphone, laboratory standard microphone, long-term stability, microphone calibration, working standard microphone

## Abstract

The devices calibrated most frequently by the acoustical measurement services at the National Institute of Standards and Technology (NIST) over the 50-year period from 1963 to 2012^1^ were one-inch condenser microphones of three specific standard types: LS1Pn, LS1Po, and WS1P. Due to its long history of providing calibrations of such microphones to customers, NIST is in a unique position to analyze data concerning the long-term stability of these devices. This long history has enabled NIST to acquire and aggregate a substantial amount of repeat calibration data for a large number of microphones that belong to various other standards and calibration laboratories. In addition to determining microphone sensitivities at the time of calibration, it is important to have confidence that the microphones do not typically undergo significant drift as compared to the calibration uncertainty during the periods between calibrations. For each of the three microphone types, an average drift rate and approximate 95 % confidence interval were computed by two different statistical methods, and the results from the two methods were found to differ insignificantly in each case. These results apply to typical microphones of these types that are used in a suitable environment and handled with care. The average drift rate for Type LS1Pn microphones was −0.004 dB/year to 0.003 dB/year. The average drift rate for Type LS1Po microphones was −0.016 dB/year to 0.008 dB/year. The average drift rate for Type WS1P microphones was −0.004 dB/year to 0.018 dB/year. For each of these microphone types, the average drift rate is not significantly different from zero. This result is consistent with the performance expected of condenser microphones designed for use as transfer standards. In addition, the values that bound the confidence intervals are well within the limits specified for long-term stability in international standards. Even though these results show very good long-term stability historically for these microphone types, it is expected that periodic calibrations will always be done to track the calibration history of individual microphones and check for anomalies indicative of shifts in sensitivity.

## 1. Introduction

The acoustical measurement services offered by the National Institute of Standards and Technology (NIST) provide the top of the traceability chain for the private and public sectors in the U.S. These sectors perform large numbers of secondary and further calibrations and measurements that support U.S. interests related to commerce, defense, product conformance, health, and safety. Standard microphones calibrated by NIST are used as references to calibrate other microphones and widely used sound calibrators, which apply known sound pressures to calibrate acoustical measuring devices and systems. Calibrations of standard microphones and acoustical calibrators done by NIST enable its customers and organizations that utilize their services to perform accurate and traceable measurements concerned with hearing conservation and testing, aircraft noise, noise regulation enforcement, acoustical test and measurement equipment, and auditory research.

Calibration services for condenser microphones designed for use as transfer standards began at NIST in 1945 when the organization was known as the National Bureau of Standards (NBS). By the end of 2012, more than 300 condenser microphones had been calibrated, many of them multiple times throughout the years, and the results conveyed to customers in 1,325 calibration reports. Due to its long history of providing calibrations of one-inch condenser microphones to customers, NIST is in a unique position to analyze data concerning the long-term stability of such microphones. This long history has enabled NIST to acquire and aggregate a substantial amount of repeat calibration data for a large number of microphones that belong to various other standards and calibration laboratories.

The condenser microphone uses variations in capacitance between a flexible diaphragm and a fixed electrode to measure the motion of the diaphragm in response to incident sound. Calibration of a standard microphone determines the modulus of its pressure sensitivity, which is the ratio of microphone open-circuit output voltage to the sound pressure uniformly distributed over the diaphragm surface. Microphones used as laboratory standards are routinely calibrated periodically to verify and track sensitivity over time and to develop a calibration history for each individual microphone. Information regarding the selection of calibration intervals is provided elsewhere [1–4].

In addition to determining microphone sensitivities from calibrations done at particular times, it is important to have confidence that microphones are extremely stable and do not typically undergo significant drift as compared to the calibration uncertainty during the intervals between calibrations when used under suitable environmental conditions and handled with care. Relaxation of the diaphragm tension, which leads to changes in the sensitivity, occurs at a fast rate after initial tensioning during manufacturing then slows exponentially with time. Artificial aging done at 150 °C after the final diaphragm tensioning during manufacturing decreases the tension and provides very good long-term stability by significantly slowing any further relaxation at room temperature [5–7]. Predictions for long-term stability at room temperature on the order of 1 dB per several hundred years have been derived from measurements done for microphones at higher temperatures up to 175 °C [5–7]. The values determined were consistent with calibration data obtained over two decades at the manufacturer’s laboratory for eight primary standard microphones that were permanently stored there at room temperature; within the experimental errors no significant change was observed in the microphone sensitivities over the measurement period [7]. International standards specify a long-term stability coefficient at room temperature of less than 0.02 dB per year for laboratory standard microphones [8], and less than 0.03 dB per year for working standard microphones [9].

The design of the condenser microphone was proposed in 1881 by Dolbear, who recognized that the same device also could be used as a source of sound [10]. Its relative insensitivity precluded extensive use until electronic amplification became available. A practical version was reported in 1917 by Wente, who recognized the potential to control the variation of sensitivity with sound frequency by tailoring various mechanical design features [11].

The next 20 years saw the development of the thermophone, a calculable source of sound for calibrating microphones [12], substantial expansion of the theory underlying microphone calibrations [13–17], and the development of improved condenser microphone designs suitable for mass production [18,19]. The commonly used calibration methods required independent knowledge of the magnitude of the applied sound pressure, based either on independent (non-microphonic) measurements [10], or on the use of a calculable source [12]. These methods could be tedious and subject to the effects of compounding errors – calculating the output pressure of a thermophone required numerical values for 21 parameters [17].

In 1929, Ballantine devised a “method of three electrophones” [20], by which the sensitivity of each microphone of a set of three could be determined from the results of three pairwise electrical measurements, provided that at least one microphone could be used both as a source and receiver. Reciprocity methods were pursued independently by MacLean, who developed a similar method for closed couplers [21], and by Cook, who in 1940 demonstrated the calibration of condenser microphones by a reciprocity method requiring the measurement of only five parameters [22].

During the 1940s, the reciprocity method was refined through a cooperative effort that involved NBS [23]. By 1949, this work had led to the adoption of the reciprocity technique as the American standard method for the pressure calibration of laboratory standard microphones [24]. Since then, improved versions of the reciprocity technique [25,26] have continued to serve this purpose.

Informal NBS microphone calibration services [27] were available before 1940. Surviving records at NIST show that formal microphone calibration services were first offered in 1945, and that 140 calibration reports had been issued by the end of March 1955. By the end of 2012, the number of reports issued was 1,325, for an average rate of 19.5 reports per year.

## 2. Selection of Microphone Data Sets

This article considers the particular microphone types submitted for calibration in quantities sufficient for statistical analysis. These are the one-inch microphone types designated LS1Pn and LS1Po designed for use as a laboratory standard [8], and the one-inch microphone type designated WS1P designed for use as a working standard [9].

The data analyzed for this article are the results of reciprocity-based comparison calibrations which currently use Type LS1Pn microphones as primary standards that are periodically calibrated using the reciprocity method. A primary standard microphone is used to transmit sound into a two port cylindrical coupler into which the microphone under calibration is also installed and serves as the receiver microphone. The application of reciprocity theory allows the sensitivity of the receiver microphone to be calculated from the calibration constants of the primary standard microphone, properties of the gas inside the coupler, and a voltage ratio that is readily determined using a step attenuator.

Microphone sensitivity is expressed using the ratio of SI derived units V/Pa, but is customarily stated as a level in decibels (dB) relative to 1 V/Pa. In this article, each microphone calibration data set constitutes a list of values of pressure sensitivity for the 18 sine wave frequencies shown in Table 1, which also shows expanded uncertainties [28] with coverage factor *k* = 2.

An electronic database maintained at NIST contains the results of all microphone calibrations performed after January 1, 1963. The data analyzed in this article were selected from the data sets for 771 calibration reports for the 152 laboratory standard microphones and 130 working standard microphones that were submitted for calibration during the 50-year interval between January 1, 1963 and December 31, 2012.

In the interests of statistical robustness, data sets were selected according to these criteria:

1. Each data set covered the 18 test frequencies shown in Table 1.
2. Data sets were available for at least ten microphones of the same model.
3. For each microphone, the time spanned by the available data sets exceeded 5 years.

These selection criteria are met by 484 data sets for 76 microphones. Of these microphones, 32 were Bruel and Kjaer Model 4160^2^ Type LS1Pn microphones, 20 were Western Electric Model 640AA Type LS1Po microphones, and 24 were Bruel and Kjaer Model 4144 Type WS1P microphones. Each microphone model will be designated by its type hereinafter.

## 3. Statistical Analysis

Data analysis began with aggregation of the selected data sets for a given microphone type. The aggregated data were sorted by microphone serial number, test frequency, and test date to create a data block showing the results of all tests of each microphone. Each data block listed values of sensitivity *M* (in V/Pa) for each test frequency in ascending order of test date. For each test frequency, for all but the first test date, we then calculated *dM*, the fractional change in sensitivity since the first test, and *dt*, the elapsed time *t* in years since the first test, and the drift rate *dM*/*dt* in percent per year.

For each of the three microphone types, average drift rates were computed for each microphone, averaging all data over both frequency and time. Each microphone average is interpreted as a measured value of a random draw from the population of true drift rates typical of all microphones of the same type that are used under suitable environmental conditions and handled with care. The distribution of these microphone averages was then studied to select appropriate statistical methods for quantifying the average drift rate for the population of microphones of each type.

One way to assess the consistency of a set of samples with an underlying normal probability distribution is to examine the normal probability plot of the data. A normal probability plot displays on the vertical axis the values of the samples, sorted from smallest to largest and displays on the horizontal axis the associated theoretical quantiles obtained from a standard normal distribution. The standard normal distribution is a normal or Gaussian distribution with mean µ = 0 and variance σ^2^ = 1. When the data are distributed approximately like random draws from a normal distribution, the points in the normal probability plot will lie roughly along a straight line.

Sometimes a best-fit line is added to a normal probability plot as a reference as well. If the data are normally distributed, the y-axis intercept of the line will estimate the mean of the data and the slope of the line will estimate the standard deviation of the data.

**Fig. 1:**
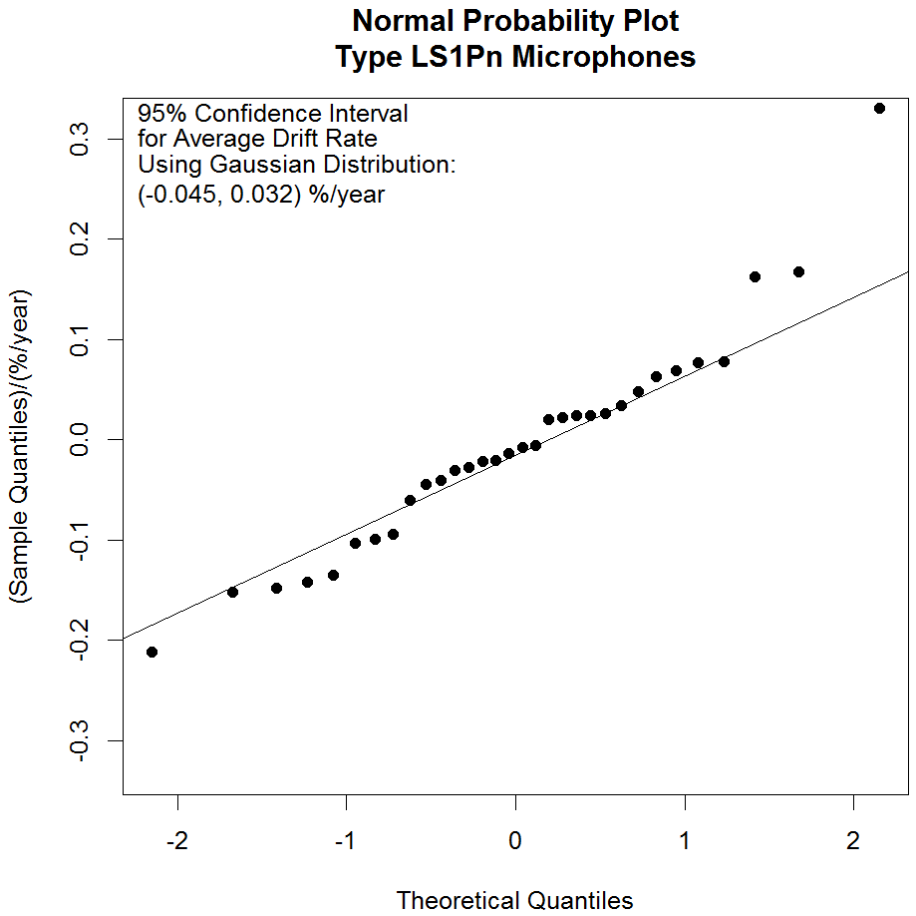
Normal probability plot of the sample means of the LS1Pn microphone drift rates.

Figure 1 shows a normal probability plot of the 32 mean microphone drift rates for the LS1Pn microphones. The plotted quantities were computed using the R environment for statistical computing and graphics [29]. The formula used to compute the theoretical quantiles is

$$z_{i}=\Phi^{-1}\left(\frac{i-0.5}{32}\right)\;\text{for}\;i=1,\ldots ,32$$ (1)

where *z_i_* is the *i*^th^ theoretical quantile and Ф^−1^ is the inverse cumulative distribution function for the standard normal distribution. The reference line shown in the plot is a line fit to the interquartile range of the data. Looking at Fig. 1, we conclude that the microphone means are distributed approximately normally since the points do fall approximately along a straight line.

Based on the assumption of normality, an approximate 95 % confidence interval for the mean drift rate of Type LS1Pn microphones can be computed using the standard approach based on the Student’s *t* distribution

$$\bar{y}-t_{1-\alpha /2,v}\frac{s}{\sqrt{n}}\leq \mu \leq \bar{y}+t_{1-\alpha /2,v}\frac{s}{\sqrt{n}}$$ (2)

where *µ* is the true mean drift rate for type LS1Pn microphones, $\bar{y}$ is the sample mean of the *n* individual microphone mean values, *s* is the sample standard deviation of the individual microphone means, and *t*_1−_*_α/_*_2,_*_ν_* is the 1−*α*/2 quantile from the Student’s *t* distribution with *v* degrees of freedom. For this study, *n* = 32, 1−*α*/2 = 0.975, and *v* = 31.

The numeric results for the Student’s *t* confidence interval are given in the upper left corner of Fig. 1 and indicate that the average drift rate for the population of Type LS1Pn microphones lies between −0.045 %/year and 0.032 %/year with 95 % confidence. The fact that this interval includes zero indicates that the population drift rate is not significantly different from zero.

Because some judgment is required to conclude that the mean drift rates of the individual microphones are normally distributed, a confidence interval for the mean of the population of microphones was also computed using a non-parametric technique, known as the bootstrap [30], for comparison. Unlike the confidence interval based on the Student’s *t* distribution, the bootstrap result does not depend on specific distributional assumptions about the data to obtain an approximate confidence interval. The bootstrap is carried out by randomly resampling with replacement from the original set of data, the 32 microphone means in this case, to create a large number of “new” data sets referred to as bootstrap samples. When the original sample set is large enough, the bootstrap samples will approximate samples drawn from the distribution of interest (i.e., the true, unknown distribution underlying the original data).

**Fig. 2:**
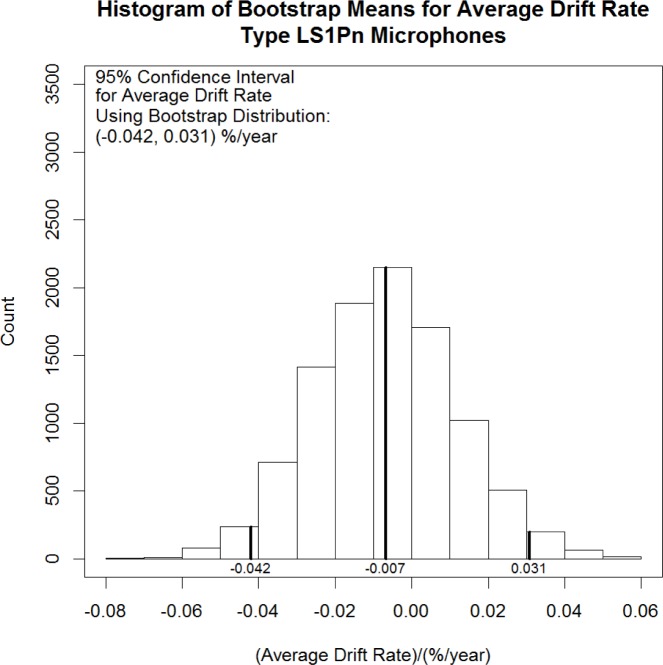
Histogram of the approximate sampling distribution for the average drift rate of Type LS1Pn microphones obtained using the bootstrap. Bold vertical lines show the 95 % confidence limits and the bootstrap mean value −0.007 %/year.

Applying the statistic of interest (the mean in this case) to the bootstrap samples, one obtains an approximation of the sampling distribution of the statistic applied to the real data based on its actual underlying distribution. Different methods, such as computing the appropriate quantiles of the sampling distribution, can then be used to obtain approximate confidence intervals for the expected value of the statistic of interest. Figure 2 shows the result from a bootstrap analysis of the LS1Pn microphone data using 10 000 bootstrap samples of size *n* = 32.

An approximate 95 % confidence interval for the average drift rate of Type LS1Pn microphones based on the bootstrap analysis is shown in the upper left corner of Fig. 2. The similarity of the bootstrap results, which suggest an average drift rate of −0.042 %/year to 0.031 %/year, to the results based on the assumption of normality are taken to confirm that the assumption of normality is reasonable for the LS1Pn microphone data.

**Fig. 3:**
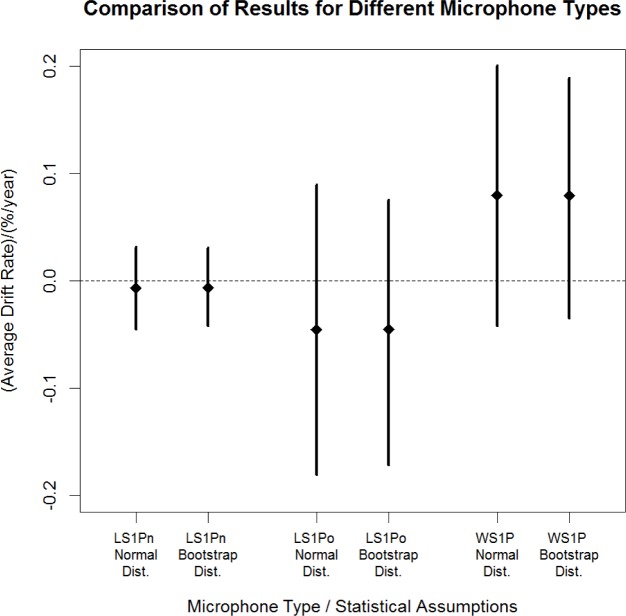
Approximate 95 % confidence intervals produced using different statistical analyses for different microphone types.

Average drift rates and approximate 95 % confidence intervals were also calculated for the LS1Po and WS1P microphone data using both Student’s *t* analysis based on the assumption of normality, and the distribution-independent bootstrap analysis. The results for the three microphone types are shown in Fig. 3. For each microphone type, the similarity of the two confidence intervals indicates that the assumption of normality is reasonable. The upper and lower limits of the confidence intervals indicate that the average drift rates are not significantly different from zero for any of the three types of microphones using either method.

The numeric results corresponding to the Student’s *t* confidence interval shown for each microphone type in Fig. 3 are given in Table 2. Ranges of average drift rates are shown both in percent per year and decibels per year.

## 4. Conclusion

Due to its long history of providing one-inch condenser microphone calibrations to customers, NIST is in a unique position to analyze data concerning the long-term stability of these devices. This long history has enabled NIST to acquire and aggregate a substantial amount of repeat calibration data for a large number of microphones that belong to various other standards and calibration laboratories.

Data sets have been analyzed for 32 Type LS1Pn microphones, 20 Type LS1Po microphones, and 24 Type WS1P microphones submitted more than once for calibration during the 50 calendar years 1963 to 2012. Each microphone type was represented by at least 18 specimens calibrated during an interval of at least 5 years within an overall span of at least 32 years.

For each microphone type, an average drift rate and approximate 95 % confidence interval were computed by two different statistical methods, and the results from the two methods were found to differ insignificantly in each case. These results apply to typical microphones of these types that are used in a suitable environment and handled with care. The average drift rate for Type LS1Pn microphones was −0.004 dB/year to 0.003 dB/year. The average drift rate for Type LS1Po microphones was −0.016 dB/year to 0.008 dB/year. The average drift rate for Type WS1P microphones was −0.004 dB/year to 0.018 dB/year. For each of these microphone types, the average drift rate is not significantly different from zero. This result is consistent with the performance expected of condenser microphones designed for use as transfer standards. In addition, the values that bound the confidence intervals are well within the limits specified for long-term stability in international standards [8,9]. Even though these results show very good long-term stability historically for these microphone types, it is expected that periodic calibrations will always be done to track the calibration history of individual microphones and check for anomalies indicative of shifts in sensitivity.

## Figures and Tables

**Table 1:** Frequencies and expanded uncertainties for reciprocity-based comparison calibrations of one-inch condenser microphones. Microphone sensitivity is expressed using the ratio of SI derived units V/Pa, but is customarily stated as a level in decibels (dB) relative to 1 V/Pa.

Frequency (Hz)	Sensitivity Level Expanded *k* = 2 Uncertainty (dB)	Sensitivity elative Expanded *k* = 2 Uncertainty (%)
50	0.08	0.93
100	0.08	0.93
200	0.08	0.93
300	0.08	0.93
500	0.08	0.93
700	0.08	0.93
1000	0.08	0.93
1500	0.08	0.93
2000	0.08	0.93
2500	0.08	0.93
3000	0.08	0.93
4000	0.08	0.93
5000	0.09	1.04
6000	0.09	1.04
7000	0.09	1.04
8000	0.26	3.04
9000	0.26	3.04
10000	0.26	3.04

**Table 2:** Results of uncertainty analysis used to establish approximate 95 % confidence intervals for the average drift rates of different microphone types.

Microphone Type	Average Drift Rate %/year	Standard Uncertainty of Average Drift Rate %/year	Degrees of Freedom	Coverage Factor from Student’s *t* Distribution	Expanded Uncertainty of Average Drift Rate %/year	Average Drift Rate Range %/year	Average Drift Rate Range dB/year
LS1Pn	−0.007	0.0190	31	2.040	0.039	−0.046 to 0.032	−0.004 to 0.003
LS1Po	−0.045	0.0648	19	2.093	0.136	−0.181 to 0.091	−0.016 to 0.008
WS1P	0.080	0.0588	23	2.069	0.122	−0.042 to 0.202	−0.004 to 0.018

## Footnotes

2 Certain commercial equipment, instruments, or materials are identified in this paper to foster understanding. Such identification does not imply recommendation or endorsement by the National Institute of Standards and Technology, nor does it imply that the materials or equipment identified are necessarily the best available for the purpose.
